# Distinct spectrum of microRNA expression in forensically relevant body fluids and probabilistic discriminant approach

**DOI:** 10.1038/s41598-019-50796-8

**Published:** 2019-10-04

**Authors:** Shuntaro Fujimoto, Sho Manabe, Chie Morimoto, Munetaka Ozeki, Yuya Hamano, Eriko Hirai, Hirokazu Kotani, Keiji Tamaki

**Affiliations:** 1 Department of Forensic Medicine, Kyoto University Graduate School of Medicine, Yoshida-Konoe-cho, Sakyo-ku, Kyoto, 606-8501 Japan; 2 Forensic Science Laboratory, Kyoto Prefectural Police Headquaters, 85-3, 85-4, Yabunouchi-cho, Kamigyo-ku, Kyoto, 602-8550 Japan

**Keywords:** miRNAs, Molecular medicine

## Abstract

MicroRNA is attracting worldwide attention as a new marker for the identification of forensically relevant body fluids. A probabilistic discriminant model was constructed to identify venous blood, saliva, semen, and vaginal secretion, based on microRNA expression assessed via RT-qPCR. We quantified 15 candidate microRNAs in four types of body fluids by RT-qPCR and found that miR-144-3p, miR-451a-5p, miR-888-5p, miR-891a-5p, miR-203a-3p, miR-223-3p and miR-1260b were helpful to discriminate body fluids. Using the relative expression of seven candidate microRNAs in each body fluid, we implemented a partial least squares-discriminant analysis (PLS-DA) as a probabilistic discriminant model and distinguished four types of body fluids. Of 14 testing samples, 13 samples were correctly identified with >90% posterior probability. We also investigated the effects of microRNA expression in skin, semen infertility, and vaginal secretion during different menstrual phases. Semen infertility and menstrual phases did not affect our body fluid identification system. Therefore, the selected microRNAs were effective in identifying the four types of body fluids, indicating that probabilistic evaluation may be practical in forensic casework.

## Introduction

Body fluid identification is crucial in forensic casework for the reconstruction of the criminal activity. Body fluid identification may indicate how the sample was left at the crime scene^1–4^. For example, saliva may have been deposited by chatting while semen may be attributable to sexual assault. Currently, morphological and enzymatic/immunological tests are performed on samples. However, these methods are difficult to apply successfully to degraded samples^2^. In addition, the sample volumes are often very small and may not suffice for the multiple tests which may be required to distinguish body fluids^2^.

Recently, RNA-based body fluid identification system has been developed. Certain messenger RNAs (mRNA) are expressed in a cell-specific manner ^5–24^ and this property has been exploited to establish mRNA-based body fluid identification systems such as ‘Cell typer’^14,16,17,25^. This system is applicable to various body fluids including the venous blood, saliva, nasal secretion, semen, vaginal secretion, menstrual blood and skin and requires no special instruments in the forensic laboratory. In forensic casework, mRNA may be remained in dried samples even if the samples are aged^1^, but some studies have reported that the expression of mRNA is affected by various environmental factors^26–28^.

In contrast, microRNAs are small molecules (17–22 bases) resistant to environmental factors because they are stored in exosomes^29^, they have no splicing variants unlike mRNA and their expression also follows a cell-specific pattern^30,31^. Recent studies have reported that several microRNAs are highly expressed in forensically relevant body fluids^32–47^ and can roughly distinguish semen, blood and epithelial cells. However, it is difficult to discriminate fluids with similar traits such as saliva and vaginal secretion. Sauer *et al*.^42^ proposed linear discriminant analyses for microRNA-based body fluid identification. This method is easy, systematic and comprehensible but their models could only distinguish between two body fluids at a time. Recently, Dørum *et al*.^47^ reported probabilistic discrimination of body fluid based on microRNA sequencing and their method is highly effective for probabilistic discrimination with the disadvantages of cost and time required. It is preferable that irrelevant factors, such as skin contamination and donor traits, do not interfere with body fluid identification because they do not elucidate how the samples were left at the crime scene or who left them there^2^. However, the actual effects of microRNA expression in skin and individual characteristics on microRNA expression have not yet been verified.

In the present study, microRNAs were quantified by RT-qPCR in forensically relevant body fluids including the venous blood, semen, saliva and vaginal secretion. The aim was to establish a microRNA-based body fluid identification system based on probabilistic discrimination. Furthermore, we investigated the effects of microRNA expression in skin on body fluid identification as well as specific donor traits such as seminal infertility and vaginal secretion changes with menstrual phase.

## Results

### Selection of candidate RNA based on amplification success and efficiency

Before comparing microRNA expression levels in the body fluids, the RT-qPCR amplification efficiency of candidate microRNAs and three reference RNAs that were selected in our previous study^48^ was determined for all samples. Of the 15 candidate RNAs (Table 1), four candidates were not amplified at all, namely, miR-200c-3p and miR-658 (saliva), and miR-124-3p and miR-3685 (vaginal secretion) (Table 1). In addition, miR-205 (saliva) showed a low amplification efficiency (1.50; Table 1). Therefore, we excluded these microRNAs because they were not suitable for our detection system and used the ten remaining candidates (miR-16-5p, miR-144-3p and miR-451a-5p for venous blood; miR-203a-3p and miR-223-3p for saliva; miR-10a-5p, miR-888-5p and miR-891a-5p for semen; miR-155-5p and miR-1260b for vaginal secretion) in the subsequent analyses.

**Table 1:** Candidate microRNAs and amplification efficiency in qPCR, analysed by LinRegPCR.

Body fluid	microRNA	miRBase Accession ID	Amplification efficiency	References
Venous blood	miR-16-5p	MIMAT0000069	1.86	^32^
	miR-144-3p	MIMAT0000436	1.85	^35^
	miR-451a-5p	MIMAT0001631	1.90	^32^
Saliva	miR-200c-3p	MIMAT0000617	—	^36, 46^
	miR-203a-3p	MIMAT0000264	1.97	^36^
	miR-205-5p	MIMAT0000266	1.50*	^32^
	miR-223-3p	MIMAT0000280	1.95	^41^
	miR-658	MIMAT0003336	—	^32^
Semen	miR-10a-5p	MIMAT0000253	1.86	^35^
	miR-888-5p	MIMAT0004916	1.87	^37^
	miR-891a-5p	MIMAT0004902	1.90	^37^
Vaginal secretion	miR-124-3p	MIMAT0000422	—	^32^
	miR-155-5p	MIMAT0000646	1.96	^42^
	miR-1260b	MIMAT0015041	1.98	^37^
	miR-3685	MIMAT0018113	—	^42^
Reference RNA	5S-rRNA		1.92	^48^
	miR-484	MIMAT0002174	1.92	^48^
	miR-92a-3p	MIMAT0000092	1.85	^48^

*Low amplification efficiency; -: amplification failure.

### Comparison of microRNA expression among five body fluids

**Figure 1:**
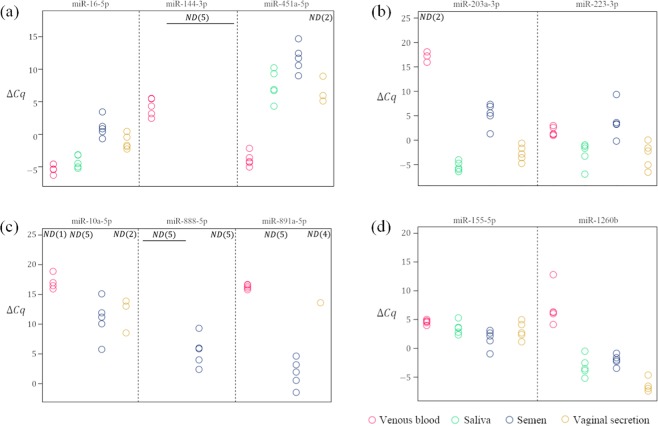
Candidate microRNAs expression in four types of body fluids. (**a**) Venous blood-relevant microRNAs miR-16-5p, miR-144-3p, and miR-451a-5p; (**b**) saliva-relevant microRNAs miR-203a-3p, and miR-223-3p; (**c**) semen-relevant microRNAs miR-10a-5p, miR-888-5p, and miR-891a-5p; (**d**) vaginal secretion-relevant microRNAs miR-155-5p, and miR-1260b. Red circles: venous blood; green circles: saliva; blue circles: semen; yellow circles: vaginal secretion (all n = 5). ‘Not detected’ is abbreviated ND. Number within brackets represents the number of ND samples.

Figure 1 shows the expression levels of ten microRNAs in each type of body fluid. The microRNAs that were relevant for venous blood, miR-16-5p, miR-144-3p and miR-451a-5p, were highly expressed in venous blood (Figure 1a). The miR-16-5p was highly expressed in all target body fluids whereas miR-144-3p was expressed only in the venous blood and miR-451a-5p showed higher expression in venous blood than that in other body fluids. When the venous blood discrimination threshold was set to a certain value such as ΔCq = 10 for miR-144-3p and 0 for miR-451a-5p in our dataset, the fluid type was easily distinguished.

The candidate microRNAs for saliva, miR-203a-3p and miR-223-3p, were highly expressed in both the saliva and vaginal secretion (Figure 1b). Hence, it was difficult to qualitatively discriminate between saliva and vaginal secretion using these microRNAs. Nevertheless, this technique could effectively discriminate saliva from venous blood and semen.

The microRNA candidates for semen, miR-10a-5p, miR-888-5p and miR-891a-5p, were highly expressed in semen. The miR-10a-5p was detected in venous blood and vaginal secretion at the same expression level as in semen. The latter two were detected at low expression levels or not detected in other body fluids (Figure 1c). Therefore, miR-888-5p and miR-891a-5p were effective for semen identification.

The microRNAs miR-155-5p and miR-1260b were detected in vaginal secretion (Figure 1d). However, the former was expressed at the same level in all body fluids and was not specific to vaginal secretion whereas the latter was highly expressed in vaginal secretion. On the other hand, it was also highly expressed in semen and saliva. Consequently, it is difficult to identify vaginal secretion using miR-1260b expression alone, but combining the other target microRNAs, such as miR-203a-3p and miR-888-5p, would be effective for identification of vaginal secretion.

We identified seven effective microRNAs (miR-144-3p and miR-451a-5p for venous blood, miR-203a-3p and miR-223-3p for saliva, miR-888-5p and miR-891a-5p for semen and miR-1260b for vaginal secretion) and used them in subsequent examinations.

### Effects of microRNA expression in skin, semen infertility and vaginal secretion during different menstrual phases

**Figure 2:**
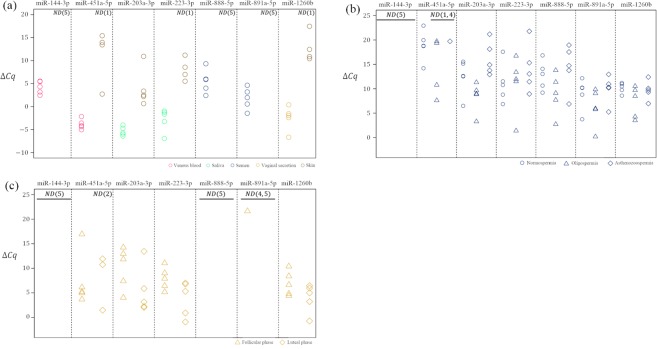
Effects of microRNA expression in skin, semen infertility and different menstrual phases on selected microRNA expression. (**a**) Effects of microRNA expression in skin on body fluid identification. Brown circles: skin; red circles: venous blood; green circles: saliva; blue circles: semen; yellow circles: vaginal secretion. (**b**) Effects of semen infertility compared among normospermic (circles), oligospermic (triangles) and asthenospermic (rectangles). (**c**) Relative microRNA expression in vaginal secretion between follicular (triangles) and luteal (rectangles) phases. N = 5 for all samples. ‘Not detected’ is abbreviated ND. Number within brackets represents the number of ND samples.

In forensic practice, skin is sometimes present in crime scene samples and we hoped that expression of our target microRNAs in skin would not disturb the identification of the four types of body fluids. We investigated the effects of microRNA expression in skin by comparing the expression levels of each candidate microRNA in skin samples with those in each body fluid. Several candidate microRNAs were expressed at low levels or not at all in skin (Figure 2a). However, miR-203a-3p, miR-223-3p and miR-1260b (saliva or vaginal secretion) were detected in four out of five skin samples. In one skin sample, miR-451a-5p (venous blood) was highly expressed. Therefore, the expression levels of miR-451a-5p, miR-203a-3p, miR-223-3p and miR-1260b could be affected by microRNA expression in skin if a biological fluid was swabbed from skin, but none of the microRNAs in skin were not expressed at similar levels to those of the target body fluids.

To determine the effect of semen infertility, we collected oligospermic semen (three out of five were azoospermic) and asthenospermic semen and evaluated the expression of each candidate microRNA (Figure 2b). Semen-relevant microRNAs (miR-888-5p and miR-891a-5p) were detected at the same expression levels in these samples as fertile semen (*P* > 0.05; Welch’s *t*-test). The other fluid-relevant microRNAs were also expressed in fertile semen at the same levels as oligospermic and asthenospermic semen (*P* > 0.05; Welch’s *t*-test).

The expression of seven candidate microRNAs was compared between the follicular and luteal phases of vaginal secretion (Figure 2c). There were no significant differences in the expression level of any of the candidate microRNAs between the follicular and luteal phases of vaginal secretion (*P* > 0.05; Welch’s *t*-test).

### Probabilistic discrimination of four types of body fluids by PLS-DA

**Figure 3:**
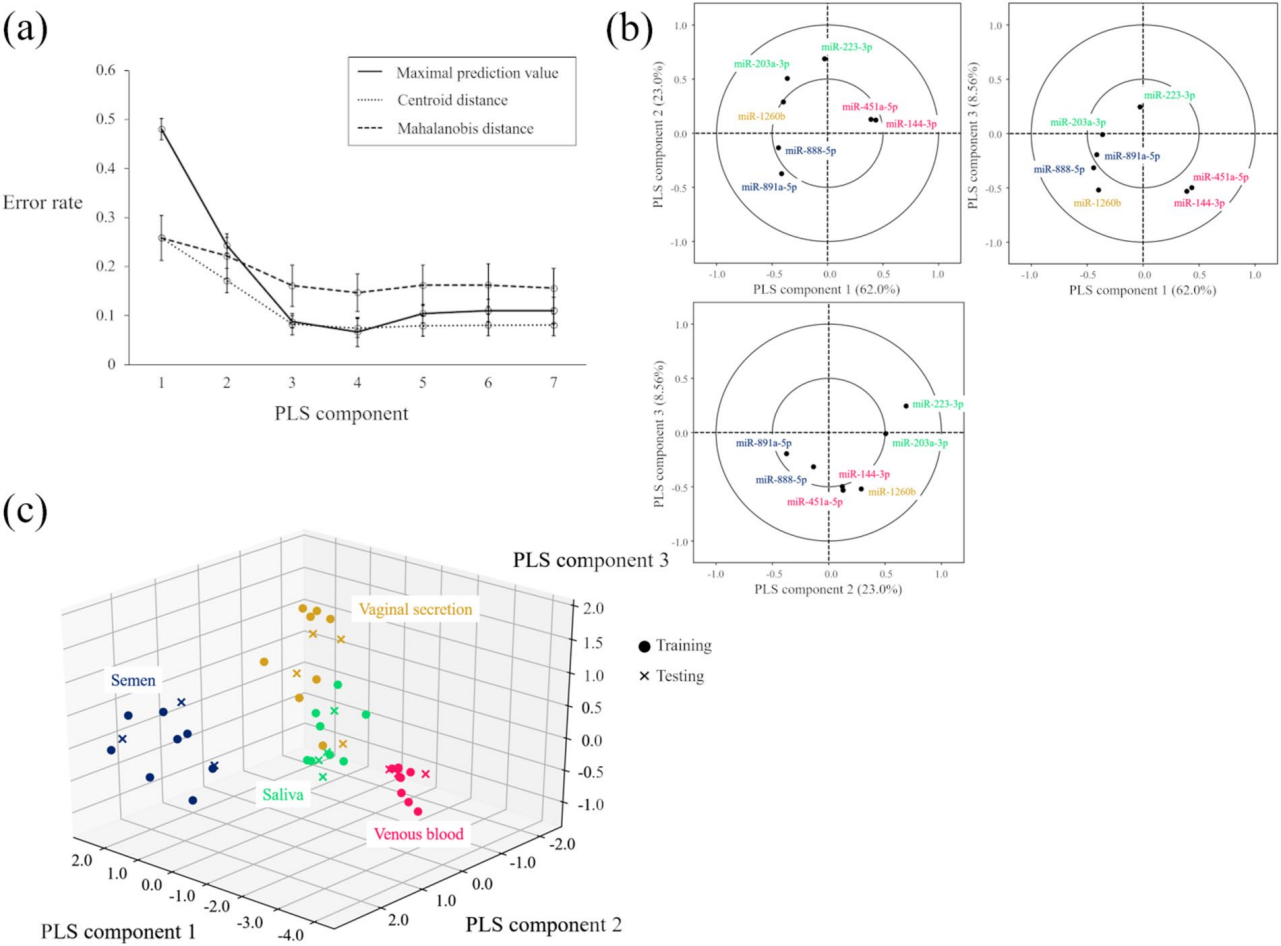
Probabilistic discrimination of four types of body fluids by seven microRNA expressions. (**a**) Optimal number of PLS components validated by five-fold cross-validation. Horizontal axis is the number of PLS components used. Vertical axis is the prediction error rate based on the training datasets. Solid lines represent the maximal prediction values. Dotted line represents results predicted by centroid distance. Dashed line represents results predicted by Mahalanobis distance. (**b**) Correlation loading plots between microRNAs and PLS components (1–3). All combinations of the three PLS components are shown. The axes of each plot describe PLS component and the percentage of explained variance. (**c**) Plots of four types of body fluid samples are shown in PLS component 1–3. Red circles: venous blood (n = 11); green circles: saliva (n = 12); blue circles: semen (n = 11); yellow circles: vaginal secretion (n = 12). Training samples indicated in ‘o’. Testing samples shown in ‘x’.

We performed probabilistic discriminant analysis by combining the ΔCq-values for seven microRNAs to identify four types of body fluids. We assigned a partial least squares-discriminant analysis (PLS-DA) to the discriminant model (Supplementary Figure 1). PLS-DA has the advantage in data analysis that the interaction between the variables can be considered by searching for latent variables (PLS components) and the PLS components are adapted to discriminate the training dataset. The model was constructed with 32 training samples and tested using 14 testing samples as shown in Materials and Methods. Figure 3a shows the optimal number of PLS components and indicates that the classification error rate decreased with increasing number of PLS components in all three distance measures (*i.e*., maximal prediction value, centroid distance and Mahalanobis distance.) However, the use of >3 PLS components did not markedly change the classification error rate. In general, the model could overfit our training data if many components were included. Therefore, we selected three PLS components to construct the probabilistic discriminant model. The correlation loading plot (Figure 3b) shows the correlation between the explanatory variables and the PLS components. For the model based on three PLS components, the correlation loading plots disclosed that miR-144-3p and miR-451a-5p, miR-203a-3p and miR-223-3p, and miR-888-5p and miR-891a-5p all had same loading value trend. Moreover, PLS component 1 was positively affected by venous blood-specific microRNAs, PLS component 2 was negatively affected by semen-specific microRNAs and PLS component 3 was positively affected by saliva-relevant microRNAs. The saliva markers were more positively correlated with PLS component 2 than with PLS component 3. Venous blood and vaginal secretion makers also positively correlated with PLS component 2, but they had negative effects in PLS component 3. The vaginal secretion-relevant microRNA (miR-1260b) was correlated to miR-203a-3p and miR-223-3p in PLS components 1 and 2, but for PLS component 3 the saliva- and vaginal secretion-relevant microRNAs were negatively correlated. For this reason, PLS component 3 was effective at discriminating between saliva and vaginal secretion. Figure 3c reveals that the four types of body fluids were separately distributed into each category. Venous blood and semen were well separated from the other body fluids whereas saliva and vaginal secretion partially overlapped. Almost all testing samples were correctly plotted in each category, but one vaginal secretion was apparently grouped with the saliva samples.

We also assigned a posterior probability for each body fluid according to Bayes theorem by fitting the PLS components to multivariate normal distribution (Supplementary Figure 1 and Table 2). All testing samples were correctly identified by maximal probability. Venous blood and semen were correctly predicted at approximate100% posterior probability. Except for one vaginal fluid sample, saliva and vaginal secretion were predicted with >90% posterior probability. The outlying vaginal fluid showed the posterior probability 77.9% and corresponded to the sample that was apparently plotted in the saliva group (Figure 3c). The posterior odds of vaginal secretion to saliva in the sample was 3.52. Moreover, five samples of azoospermic semen were examined for proof of no effects of semen infertility in probabilistic discrimination (Supplementary Table 1). All five azoospermic semen samples were correctly identified to be semen with over 98% posterior probability.

**Table 2:** Posterior probability of testing samples assigned by PLS-DA.

Testing sample	*Prediction*			
	*Blood*	*Saliva*	*Semen*	*Vaginal secretion*
Blood 1	≈1*	≈0**	≈0**	5.12 × 10^−17^
Blood 2	≈1*	≈0**	≈0**	9.36 × 10^−15^
Blood 3	≈1*	≈0**	≈0**	7.29 × 10^−19^
Saliva 1	6.48 × 10^−23^	**0.965**	4.85 × 10^−11^	0.0346
Saliva 2	3.66 × 10^−48^	**0.982**	1.53 × 10^−5^	0.0180
Saliva 3	2.54 × 10^−52^	**0.989**	4.95 × 10^−6^	0.0106
Saliva 4	7.46 × 10^−38^	**0.990**	5.41 × 10^−7^	0.0104
Semen 1	≈0**	1.12 × 10^−14^	**0.9999999958**	4.22 × 10^−9^
Semen 2	≈0**	1.74 × 10^−7^	**0.999955**	4.50 × 10^−5^
Semen 3	≈0**	2.96 × 10^−17^	**0.99999999941**	5.91 × 10^−10^
Vaginal secretion 1	7.74 × 10^−22^	0.0633	4.31 × 10^−21^	**0.937**
Vaginal secretion 2	2.92 × 10^−19^	0.221	6.45 × 10^−8^	**0.779**
Vaginal secretion 3	1.00 × 10^−43^	0.0426	5.38 × 10^−7^	**0.957**
Vaginal secretion 4	2.48 × 10^−28^	5.38 × 10^−13^	1.03 × 10^−19^	≈1*

*Posterior probability >0.9999999999; **Posterior probability <1.0 × 10^−100^.

The four body fluid types were clearly distinguished probabilistically and the calculated probability could prove useful in the reconstruction of the criminal activity by body fluid identification.

## Discussion

The present study demonstrated that microRNAs are effective at identifying forensically relevant body fluids. Venous blood is readily identified with miR-144-3p and miR-451a-5p and semen is easily detected using miR-888-5p and miR-891a-5p. Nevertheless, saliva and vaginal secretion were not qualitatively distinguishable. Saliva and vaginal secretion have similar properties in that they contain epithelial cells, indigenous bacteria and mucus. Therefore, they are expected to express the same microRNAs at similar levels. However, miR-203a-3p and miR-223-3p in saliva and the miR-1260b in vaginal secretion were expressed more highly than they were in the other body fluids. Probabilistic discrimination, then, should effectively identify saliva and vaginal secretion.

Certain microRNAs were expressed at levels different from those reported in previous studies^32,36,42,46^. In addition, four microRNAs were not detected in all body fluids. One possible reason is the difference in the detection methods used. We used a Universal cDNA Synthesis Kit II (Exiqon, Vebaek, Denmark) for the RT reaction. It adds a poly (A) tail to the 3ʹ-terminal regions of the targets and transforms the RNAs in all samples to cDNAs. The advantage of this method is that it mitigates the influence of variability among target RNAs in the RT reaction. Certain previous studies used a stem-loop primer that reacts with each target microRNA by short-length hybridisation. This method has a higher specificity for target microRNA than ours, but the efficacy of RT reaction would be various in each target microRNA. Furthermore, it is impractical because the extracted RNA is lost by quantifying the target microRNAs every time. For quantification, the SYBR Green or TaqMan^®^ Assays (Applied Biosystems^TM^, Weiterstadt, Germany) was generally used. TaqMan^®^ Assays quantify target microRNAs more specifically than SYBR Green methods. In the present study, however, the use of locked nucleic acid (LNA) technology with amplification primers increased the specificity to the same level as that for the TaqMan^®^ Assays. To our knowledge, no studies have explored this discrepancy in microRNA detection between methods; however, certain reports including ours, have supported this finding^37,42^.

We examined the effects of microRNA expression in skin. Our results showed that miR-203a-3p, miR-223-3p and miR-1260b (saliva and vaginal secretion) were detected in four out of five skin samples. Skin contains epithelial cells, indigenous bacteria and sebum. Some of these may also appear in saliva and vaginal secretion. In the present study, body fluid samples were carefully collected to avoid contaminating them with skin. In forensic practice, however, such contamination is inevitable under certain circumstances, especially when the fluids must be sampled directly from victim body surfaces. In the identification of saliva and vaginal secretion, then, the effect of skin contamination must be considered. In addition, miR-451a-5p was detected at a high expression level in one skin sample. Four out of five skin samples expressed miR-451a-5p but its expression level in three of these samples was much lower than that of venous blood. The RNA yield was low for the skin sample highly expressing miR-451a-5p (Supplementary Table 2). Moreover, 5S-rRNA of a skin sample was expressed at comparably lower levels (Cq of the sample: 30.56 and mean of Cq of the others: 18.95). The miR-451a-5p upregulation in the skin sample may have been the result of the unstable amplification of a small amount of extracted RNA. Therefore, the stability of the RT reaction and amplification in low template samples must be verified prior to their application in forensic practice.

Moreover, we examined the effects of semen infertility on semen identification. Expression of the candidate microRNAs was not affected by semen infertility. A previous study used miR-888-5p and miR-891a-5p and reported that infertile semen affected semen identification^49^. This finding may be due to the difference in sample size (n = 144) compared with the present study (n = 10). Nevertheless, this discrepancy can also be explained by the difference in the detection systems used in these studies. The small nucleolar RNAs (SNORD24 and SNORD38B) used as reference RNAs in the previous study might have been inappropriate for semen identification. In a previous study, we found that certain semen samples did not express small nucleolar RNAs^48^. Using our detection systems, we found for our data that miR-888-5p and miR-891a-5p could be used in semen identification regardless of semen infertility. Our data showed high variation of microRNA expression in each category. The variation was mainly due to individual variances because the degree of infertility was varied and another reason could be the use of reference RNA as only 5S-rRNA for normalisation because some samples showed extremely low expression of miR-484 which was used as reference RNA in probabilistic identification of four types of body fluids.

We constructed a probabilistic discrimination model for four types of body fluids based on seven selected microRNAs. This method might have applications in the forensic casework addressed in previous reports on mRNA-based body fluid identification^24,50^. In a trial, a jury could easily understand the result of the analysis of a stain and evaluate the evidence used in crime reconstruction. Our results showed that venous blood, saliva, semen and vaginal secretion were correctly identified and predicted at high posterior probability (>90%). In addition, azoospermic semen samples were identified to be semen. Therefore, probabilistic evaluation by PLS-DA was effective for discriminating four types of body fluids despite the small number of training samples and regardless of semen infertility. However, the posterior probability for one vaginal fluid sample was substantially lower than that for the other fluids (77.9%). Moreover, the probability that it was misidentified as saliva was 22.1%. These results suggest that saliva and vaginal secretion have similar traits. At 77.9% probability, it could be difficult to conclude that a crime scene sample was vaginal fluid because the posterior odds between vaginal secretion and saliva was only 3.52. In forensic practice, a probabilistic statement may help the jury render a verdict even when the probability is low. The judgment is executed on the basis of a comprehensive estimation of criminal activity by use of body fluids, criminal investigation information and eyewitness testimony^3,4^. A previous study reported that criminal activity can be probabilistically estimated by a Bayesian network^51^. We concur with the probabilistic reconstruction of criminal activity and believe that our method can assign probabilities for body fluids via microRNA expression. Additional training samples and markers such as mRNA and DNA methylation could enhance the discriminant power of our constructed model. To apply it to actual cases, we plan to validate our probabilistic model using aged and/or degraded samples and mixed body fluids with additional training samples in future studies.

In conclusion, we identified seven microRNAs to discriminate four types of forensically relevant body fluids. We assessed forensically practical issues including microRNA expression in skin and donor characteristics such as semen infertility and menstrual cycles. We then proposed a probabilistic approach for distinguishing the body fluid samples. Our findings support the application of a microRNA-based body fluid identification system to forensic practice and we expect the system to be useful solving forensic challenges.

## Materials and Methods

### Ethical statement

All volunteers provided written informed consent. This study was approved by the ethics committee of the Graduate School of Medicine of Kyoto University (Approval No. G1053-2) and was conducted according to the principles of the Declaration of Helsinki.

### Sample preparation

Five forensically relevant body fluids (venous blood (n = 11); saliva (n = 12); semen (n = 16 including infertile semen); vaginal secretion (n = 12, divided into follicular and luteal phases); and skin (n = 5)) were collected from volunteers. Sample collection was performed by the same procedure as that used in our previous study^48^, except for the skin, which was collected by swabbing the foreheads of volunteers within 2 h after washing their faces.

Total RNA extraction and RNA yield and integrity measurements were performed by the same protocols used in our previous study^48^. RNA yield and integrity data are listed in the additional information (Supplementary Table 2).

### RT-qPCR

The 15 candidate microRNAs were selected from previous studies in which they were highly expressed in body fluids (Table 1)^32,35–37,41,42,46^. For quantitative analysis, we assigned 5S-rRNA, miR-484 and miR-92a-3p as reference RNAs and evaluated their expression in all body fluids. These were validated in our previous study^48^. To investigate microRNA expression in skin, semen infertility and different menstrual phases, 5S-rRNA was used as a reference RNA because certain samples did not express miR-484 or miR-92a-3p in our preliminary experiment (data not shown).

RT-qPCR was performed with the miRCURY LNA^TM^ Universal RT miRNA PCR System (Exiqon) in triplicates for each sample according to the manufacturer’s protocol. Template total RNA 10 ng in 10-µL reaction volume in reverse transcription reaction and 4-µL of 80-fold-diluted template cDNA was applied in qPCR. RT-qPCR quality was validated against a negative H_2_O control, a negative RT-enzyme control and a positive synthetic RNA spike-ins control (Exiqon). Melting analyses and polyacrylamide gel electrophoresis verified that no by-products were generated in the PCR assays.

Cq values were assigned with the LinRegPCR program (January 2016 version)^52,53^, which set the noise baseline and computed the Cq values and amplification efficiencies. The median of the triplicate Cq values was selected for the subsequent analyses. The exclusion criterion was set to Cq > 35. When any of the triplicate Cq values in a microRNA was >35, we determined that the microRNA was not detected in a sample (N.D.).

ΔCq were calculated as follows: ΔCq = Cq (target RNA) – Cq (reference RNA). The Cq (reference RNA) were assigned as the geometric mean of expression of three reference RNAs expression^54^ except for when examining skin samples, semen infertility and the different menstrual phases. In such cases, 5S-rRNA expression was assigned. A negative value of ΔCq for a microRNA means that it is highly expressed in a sample.

All experiments fully complied with the essential information in the MIQE guidelines^55^ and allowed reliable and unequivocal interpretation of the qPCR results.

### Assessing the effects of semen infertility and vaginal secretion at different phases

The semen and vaginal secretion samples were randomly selected so the number of samples was the same in each category (n = 5). Semen samples included normospermic semen, oligospermic semen (of which three were azoospermic) and asthenospermic semen and the vaginal secretion samples were taken from the follicular and luteal phases.

Welch’s *t*-tests were used to compare the expression levels of the selected microRNAs between normospermic and oligospermic, normospermic and asthenospermic and follicular and luteal vaginal secretions. A *P*-value <0.05 was considered statistically significant. Statistical tests were performed in R (v.3.5.1)^56^.

### Probabilistic discrimination of four types of body fluids

We performed probabilistic discrimination at two steps, PLS-DA and Bayes theorem (Supplementary Figure 1). PLS-DA has various advantages such as a smaller sample size than explanatory variables can be applied, the interaction between the variables can be considered by searching for latent variables (PLS components) and the PLS components are adapted to discriminate the training dataset. The analysis of PLS-DA was executed by the ‘mixOmics’ package in R^57,58^. Of the 46 body fluid samples, 32 (eight samples in each body fluid) were used in model building and 14 (three in venous blood and semen and four in saliva and vaginal secretion) were used in probabilistic prediction. In addition, five azoospermic semen samples were tested probabilistically for the examination of no-sperm effects. When no microRNA was detected, a conservative value of Cq = 35 was assigned to the microRNA expression in the sample. To determine the optimal number of PLS components, a five-fold cross-validation was conducted using the ‘mixOmics’ package. This procedure was repeated for every number of PLS components 1,000 times and the prediction error was calculated by maximal prediction value, centroid distance and Mahalanobis distance.

Probabilistic evaluation was performed on the basis of Bayes theorem. Based on a typical forensic approach using DNA-based personal identification, hypotheses were tested for the four body fluid types $({H}_{i}:{H}_{blood},{H}_{saliva},{H}_{semen},{H}_{vaginal})$. The results of the microRNA expression were represented as *E*. A posterior probability for the blood sample is shown below:

$${\Pr }({H}_{blood}|E)=\frac{{\Pr }({H}_{blood}){\Pr }(E|{H}_{blood})}{{\sum }_{i}{\Pr }({H}_{i}){\Pr }(E|{H}_{i})}$$

where ${\Pr }({H}_{blood}|E)$ is the posterior probability that the crime scene sample is blood given the microRNA expression in the sample and ${\Pr }({H}_{blood})$ is the prior probability. The prior probabilities are set equally $({\Pr }({H}_{blood})=0.25)$. ${\Pr }(E|{H}_{blood})$ is the likelihood that a blood sample have such a microRNA expression profile. The likelihood was assigned by fitting the PLS components of the training dataset to a multivariate Gaussian distribution for each body fluid type.

## Supplementary information

Supplementary information
