# Supplementary material for: Distinct spectrum of microRNA expression in forensically relevant body fluids and probabilistic discriminant approach
Source: Sci Rep. 2019 Oct 4;9:14332. doi: 10.1038/s41598-019-50796-8 (PMC6778116; doi:10.1038/s41598-019-50796-8)
Supplement: Supplementary file 1 — Supplementary information [file 41598_2019_50796_MOESM1_ESM.pdf]

Supplementary information

Title

Distinct spectrum of microRNA expression in forensically relevant body fluids and probabilistic discriminant approach

Authors

Shuntaro Fujimoto[1], Sho Manabe[1], Chie Morimoto[1], Munetaka Ozeki[1], Yuya Hamano[1,2], Eriko Hirai[1], Hirokazu Kotani[1], Keiji Tamaki[1,*]

Affiliations

[1] Department of Forensic Medicine, Kyoto University Graduate School of Medicine, Yoshida-Konoe-cho, Sakyo-ku, Kyoto 606-8501, Japan

[2] Forensic Science Laboratory, Kyoto Prefectural Police Headquaters, 85-3, 85-4, Yabunouchi-cho, Kamigyo-ku, Kyoto 602-8550, Japan

* Correspondence and requests for materials should be addressed to K. T. (email: ktamaki@fp.med.kyoto-u.ac.jp)

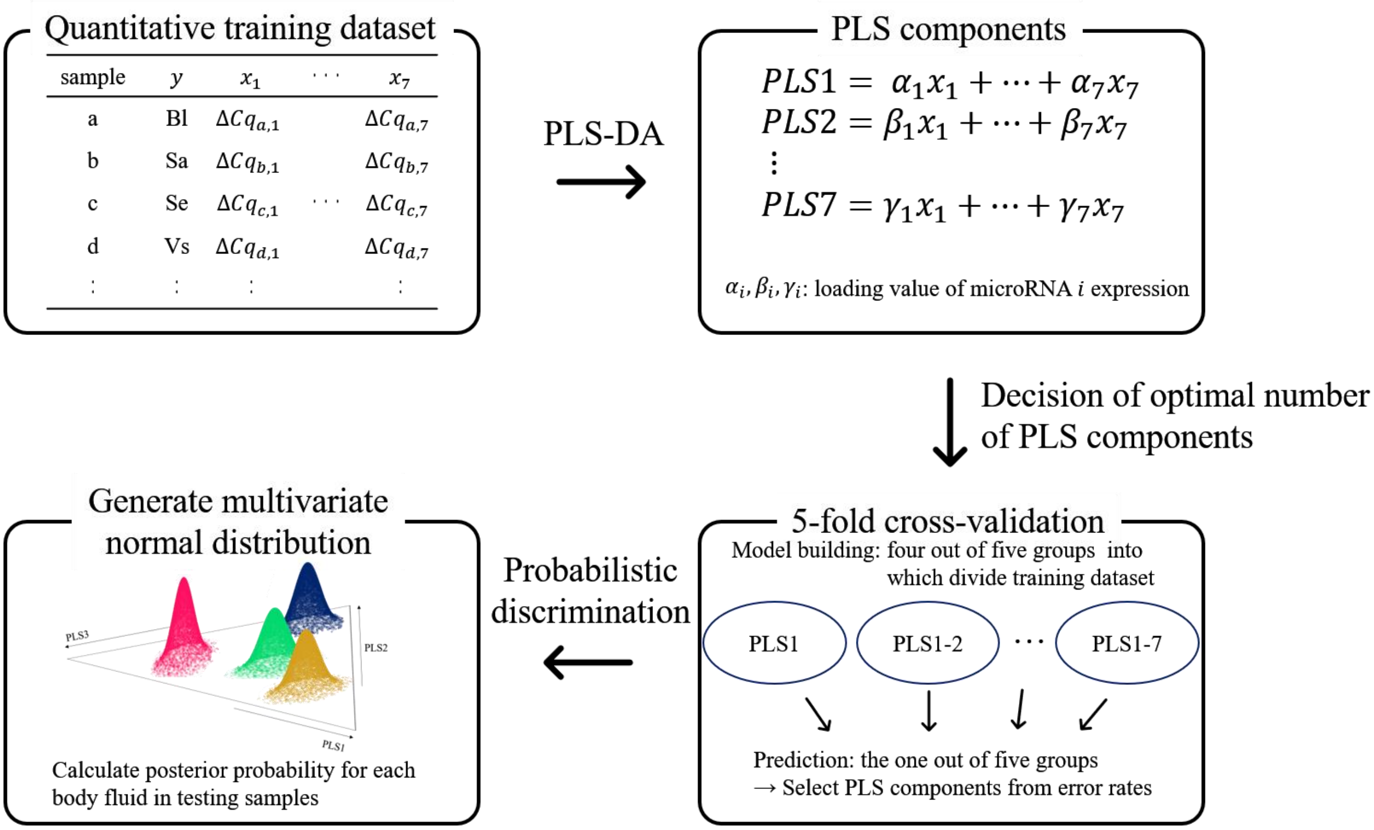

Supplementary Figure 1

Schematic representation of data analysis.

Supplementary Table 1

Posterior probability of azoospermic semen by PLS-DA.

**Azoospermic semen**	***Prediction***			
	Blood	*Saliva*	*Semen*	*Vaginal secretion*
Sample 1	≈ 0**	7.05×10^{-15}	≈ 1*	5.65×10^{-11}
Sample 2	≈ 0**	4.87×10^{-23}	≈ 1*	3.30×10^{-16}
Sample 3	≈ 0**	1.77×10^{-15}	0.983	0.166
Sample 4	≈ 0**	8.51×10^{-11}	0.999964	3.64×10^{-5}
Sample 5	≈ 0**	1.88×10^{-24}	≈ 1*	4.86×10^{-18}

*Posterior probability > 0.9999999999; **Posterior probability $< 1.0 \times 10^{-100}$

Supplementary Table 2

Total RNA quantity and integrity.

Body fluid		Total RNA (ng/μL)	RIN
Venous blood		10.2–57.0	1.7–6.5
Saliva		5.28–20.8	1.0–2.4
Semen	Normospermia	10.9–44.6	2.6–6.0
	Oligospermia	11.8-31.6	2.5–7.9
	Asthenospermia	8.36–38.1	3.0–6.6
Vaginal secretion	Follicular phase	7.36–26.0	2.2–4.9
	Luteal phase	9.92–30.8	1.0–2.7
Skin		0.85–4.96	1.0–1.4
